# Glycosylation of Human IgA Directly Inhibits Influenza A and Other Sialic-Acid-Binding Viruses

**DOI:** 10.1016/j.celrep.2018.03.027

**Published:** 2018-04-05

**Authors:** Michael A. Maurer, Larissa Meyer, Matteo Bianchi, Hannah L. Turner, Ngoc P.L. Le, Marco Steck, Arkadiusz Wyrzucki, Vanessa Orlowski, Andrew B. Ward, Max Crispin, Lars Hangartner

**Affiliations:** 1 Institute of Medical Virology, University of Zurich, Winterthurerstrasse 190, 8057 Zurich, Switzerland; 2 Department of Integrative Structural and Computational Biology, The Scripps Research Institute, 10550 North Torrey Pines Road, La Jolla, CA 92037, U.S.A; 3 Oxford Glycobiology Institute, Department of Biochemistry, University of Oxford, South Parks Road, Oxford OX1 3QU, UK; 4 Center for Biological Sciences, University of Southampton, Highfield Campus, Southampton, SO17 1BJ, UK; 5 Institute for Life Sciences, University of Southampton, Highfield Campus, Southampton, SO17 1BJ, UK

**Keywords:** immunoglobulin, antibodies, IgA, glycosylation, virus neutralization, innate immunity, influenza virus, heterosubtypic antibodies, mucosal immunity, neuraminidase

## Abstract

Immunoglobulin A (IgA) plays an important role in protecting our mucosal surfaces from viral infection, in maintaining a balance with the commensal bacterial flora, and in extending maternal immunity via breast feeding. Here, we report an additional innate immune effector function of human IgA molecules in that we demonstrate that the C-terminal tail unique to IgA molecules interferes with cell-surface attachment of influenza A and other enveloped viruses that use sialic acid as a receptor. This antiviral activity is mediated by sialic acid found in the complex N-linked glycans at position 459. Antiviral activity was observed even in the absence of classical antibody binding via the antigen binding sites. Our data, therefore, show that the C-terminal tail of IgA subtypes provides an innate line of defense against viruses that use sialic acid as a receptor and the role of neuraminidases present on these virions.

## Highlights

• Heterosubtypic IgA1 or IgA2 antibodies neutralize virus much more potently than IgG1
• Sialic acid in IgA’s C-terminal tail competes with viral receptor binding
• This may represent an innate line of defense against viral pathogens

## Graphical Abstract

**Figure:**
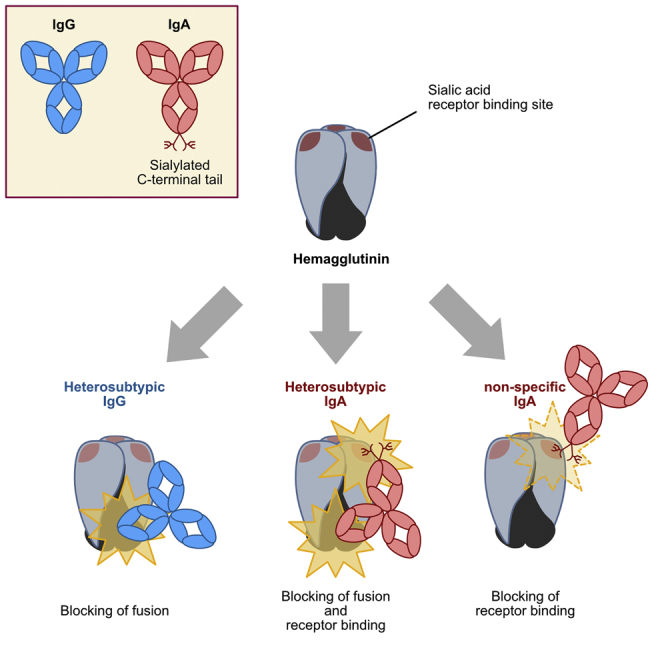

## Teaser

Vertebrate IgA molecules possess a conserved N-linked glycosylated C-terminal tail. Maurer et al. show that sialic acid found in the complex glycosylation of the C-terminal tail of human IgA1 inhibits sialic-acid-binding viruses and, therefore, may constitute an additional line of innate immunity.

## Introduction

Vaccination against influenza A viruses relies on the induction of strain-specific neutralizing immunoglobulin G (IgG) and has to be repeated annually. Rare heterosubtypic antibodies, i.e., antibodies that are able to neutralize multiple strains and subtypes of influenza virus, can be found in most individuals (Corti et al., 2010, Kohler et al., 2014) but currently cannot specifically be induced by immunization.

Immunoglobulin A (IgA) exists as monomeric molecules in serum or as dimeric secretory IgA on mucosal surfaces. Although roughly three quarters of the daily antibody production (i.e., about 3 mg) are of the IgA isotype (Delacroix et al., 1982), IgA deficiency is frequent (∼1 in 300–600 individuals) and mostly asymptomatic (Janzi et al., 2009). IgA-deficient mice exhibit an enhanced susceptibility to influenza A virus infection and display impaired T helper cell priming (Arulanandam et al., 2001). In humans and great apes, two IgA subtypes are found: IgA1 that is characterized by a 23-amino-acid (aa)-long and heavily O-glycosylated hinge region, and a conserved C-terminal tail of 19 amino acids that interacts with the J chain and secretory chain to mediate dimerization (Correa et al., 2013) and that contains a N-linked glycosylation site. IgA2 more closely resembles the IgA isotypes of other vertebrates (Rogers et al., 2008), and it also possesses a C-terminal tail, but its shorter hinge region is devoid of O-linked glycosylation.

All Ig isotypes contain complex N-linked glycosylation: while about 10% of the N-linked glycans in IgG1 contain sialic acid (Wuhrer et al., 2007) over 90% of the N-linked glycans are sialylated in IgA1. Monomeric and secretory IgA1 glycosylation of CH2 at position 263 is predominantly of the biantennary type with α2–6-linked sialic acids, while CH3 glycans at position 459 are of the triantennary type, with α2–6 and α2–3 linkage in their sialic acids (Mattu et al., 1998, Royle et al., 2003).

Recent reports also suggest that heterosubtypic IgA has a more potent antiviral activity against influenza viruses than IgG (He et al., 2015, Muramatsu et al., 2014, Yu et al., 2013). Some of these observations could be explained by immune-geography, i.e., IgA’s preferred secretion into the pulmonary lumen and increased avidity due to multimerization. Moreover, it has been shown that both IgA and secretory component are important mediators of innate immunity against various bacterial pathogens: sialic acids on secretory IgA (sIgAs) inhibit attachment of S-fimbriated *Escherichia coli* (Schroten et al., 1998), while N-linked glycosylation of secretory component (alone or as part of sIgA) has been shown to compete with *Helicobacter pylori* for receptors (Borén et al., 1993) and was shown to bind to *Escherichia coli* (de Oliveira et al., 2001, Wold et al., 1990), toxin A from *Clostridium difficile* (Dallas and Rolfe, 1998), and *Streptococcus pneumonia* (Hammerschmidt et al., 1997, Zhang et al., 2000).

To assess the impact of glycosylation and hinge length on the activity of heterosubtypic antibodies to influenza A virus, monoclonal antibodies (mAbs) 1.12 (Wyrzucki et al., 2015) and 3.1 (Wyrzucki et al., 2014) were recombinantly expressed as human IgG1, IgG3, IgA1, and IgA2 molecules, and their *in vitro* antiviral activity was characterized.

## Results

**Figure 1:**
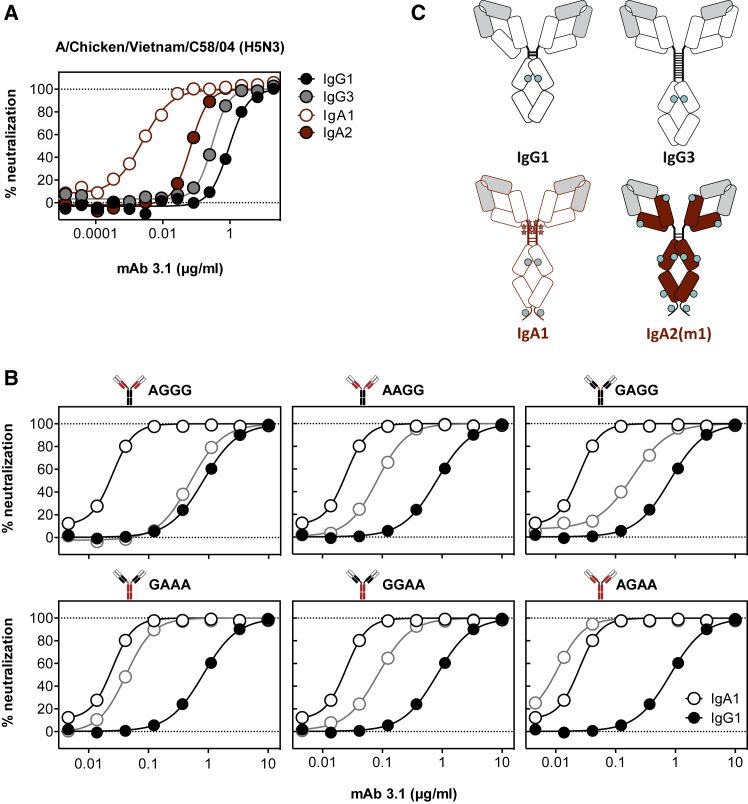
Comparison of the Neutralizing Activity of mAb 3.1 Expressed as an IgG1, IgG3, IgA1, and IgA2 Molecule (A) Neutralizing activity of monomeric 3.1 isotypes. mAb 3.1 IgA1 (red-bordered circles), IgA2 (red circles), IgG1 (black circles), and IgG3 (gray circles) against rg-A/Chicken/Vietnam/C58/2004 (H5N3). Both IgA subtypes neutralized more potently than the IgG subtypes. (B) Neutralizing activity of IgG1/IgA1 chimeric 3.1 antibodies. The four-letter code refers to the isotype origin of the CH1, hinge, CH2, and CH3, respectively. As a control, 3.1 was included as a pure IgG1 molecule (black closed circles) and an IgA1 molecule (black-bordered open circles) and plotted in each panel. The titration curves of the indicated chimeric molecules are depicted in gray-bordered open circles, while those for IgG1 and IgA1 are depicted in black and white circles, respectively. On top of each panel, the chimerism of the molecule is depicted with IgG1-derived areas in black, IgA1-derived areas in red, and the variable region in white. (C) Schematic representation of isotypes used. Variable regions are depicted as gray boxes, intermolecular disulfide bridges are depicted as horizontal lines, N-linked glycosylation are depicted as cyan circles, and O-linked glycosylation are depicted as stars.

Using isotype variants of influenza A virus-specific heterosubtypic mAbs 3.1 and 1.12, the impact of the antibody isotype on the neutralizing activity *in vitro* was assessed. While all IgG subtypes had comparable neutralizing activities, both IgA subtype monomers were 10- to 1,000-fold more potent than IgG1, depending on the mAb and isolate tested (Figures 1 and S1). Overall, the isotype-dependent activity differences were less prominent in human compared to avian isolates, and the most prominent difference in neutralization was seen with mAb 3.1 against reassortant rg-A/Chicken/Vietnam/C58/2004 (H5N3). This enhanced antiviral activity could not be attributed to the longer hinge of IgA1, as IgA2 with its shorter hinge was also more potent, while IgG_3,_ with the longest hinge, neutralized similarly to IgG1 (Figure 1). There were no differences in avidity for hemagglutinin (HA) detected for the different isotype variants (Figure S2).

**Figure 2:**
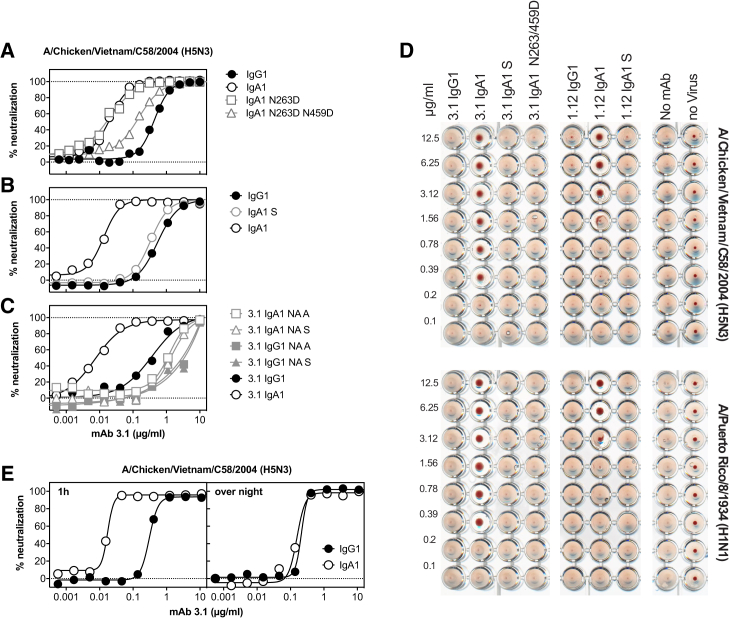
Impact of Fc Glycosylation (A) Impact of Fc glycosylation on neutralizing activity. Comparison of the neutralizing activities of wild-type IgG1, wild-type IgA1, and IgA1 in which the glycosylation consensus sequences at position 263 or 459 have been removed. (B) Comparison of the complex glycoforms of IgG1 and IgA1 and the oligomannose-type glycoform of IgA1 expressed in 293S cells (IgA S). (C) Impact of neuraminidase treatment on the neutralization of rg-A/Chicken/Vietnam/C58/2004 (H5N3). The neutralizing activity of the indicated antibodies incubated either with α2–3 neuraminidase from *Salmonella typhimurium* LT2 (NA S) or α2–3,6,8,9 neuraminidase A from *Arthrobacter ureafaciens* (NA A) was compared to that of the mock-treated IgA1 and IgG1 molecules. (D) Impact of Fc glycosylation on hemagglutination inhibition. A constant amount of the indicated virus was incubated with titrated amounts of the indicated antibodies and added to chicken erythrocytes that were then allowed to sediment at room temperature. (E) Impact of prolonged incubation with virus. Virus and titrated amounts of the indicated isotype of mAb 3.1 were incubated for either 1 hr or overnight before residual infectivity was determined.

To identify the domain of the Ig molecule responsible for this increased potency, chimeric molecules were prepared by exchanging the CH1 the hinge, and the CH2/CH3 domains from IgA1 and IgG1 with each other. The neutralizing activity of these chimeric molecules revealed that, although the hinge region has some impact on the antibody’s potency, the enhanced neutralization phenotype segregated with the CH2-CH3 portion. To address the impact of the C-terminal tail with its additional N-linked glycosylation site, variants of mAb 3.1 IgA1 were prepared in which the glycosylation sites in CH2 and CH3 (Kabat positions 263 and 459) were removed. While the non-glycosylated N263D and N263D/N459D variants expressed well, the N459D did not express. Loss of both the CH2 and CH3 glycosylation, but not of the glycosylation in CH2 alone, abolished the improved neutralizing activity (Figure 2), indicating that the N-linked glycosylation at the N459D position is essential. The requirement for the complexity of the glycosylation was assessed by expression of mAb 3.1 IgA1 in 293S cells devoid of N-acetylglucosaminyltransferase I (GnTI), which traps N-linked glycosylation at Man_5_GlcNAc_2_ (Reeves et al., 2002). The resulting oligomannose-type glycoform of 3.1 IgA1 antibody only neutralized as potently as IgG1, indicating that the increased neutralizing activity of the IgA1 isotype had its origin in the complex N-linked glycosylation in the C-terminal tail of CH3. As this glycan is reportedly sialylated (Mattu et al., 1998), and since influenza A viruses use sialic acid as a receptor, IgA1 and IgG1 of mAb 3.1 were treated either with α2–3-specific neuramindase from *Salmonella typhimurium* LT2 (NA S) or with 2-3,6,8,9-specific neuraminidase A from *Arthrobacter ureafaciens* (NA A). Both neuraminidase treatments resulted in reduced neutralizing antibody titers compared to the untreated IgA1 molecule, indicating that removal of sialic acid—in particular, that of α2–3-linked sialic acid—sufficed to reduce the potency of the IgA1 molecules considerably. The neuraminidase treatment also slightly reduced the neutralizing activity of the virtually unsialylated mAb 3.1 IgG1 molecules, suggesting additional loss of activity unrelated to the respective glycosidase activity.

To test whether C-terminal sialylation was interfering with receptor binding, hemagglutination inhibition assays were performed. Although stem-specific heterosubtypic antibodies should not inhibit hemagglutination, IgA1 of both mAb 3.1 and mAb 1.12 prevented hemagglutination by rg-A/Chicken/Vietnam/C58/2004 (H5N3) and A/Puerto Rico/8/1934 (H1N1) (Figure 2). By contrast, IgG1 or IgA1 molecules lacking complex glycosylation, i.e., IgA1S and IgA1 N263D/N459D, failed to inhibit hemagglutination (Figure 2D). Hence, receptor binding of influenza A viruses is likely competed by the complex N-linked glycans from the IgA tail. Interestingly, when antibody and virus were incubated for a prolonged period of time, the differences between IgG1 and IgA1 vanished, suggesting that the viral neuraminidase was capable of removing the antibody’s sialic acids (Figure 2E).

**Figure 3:**
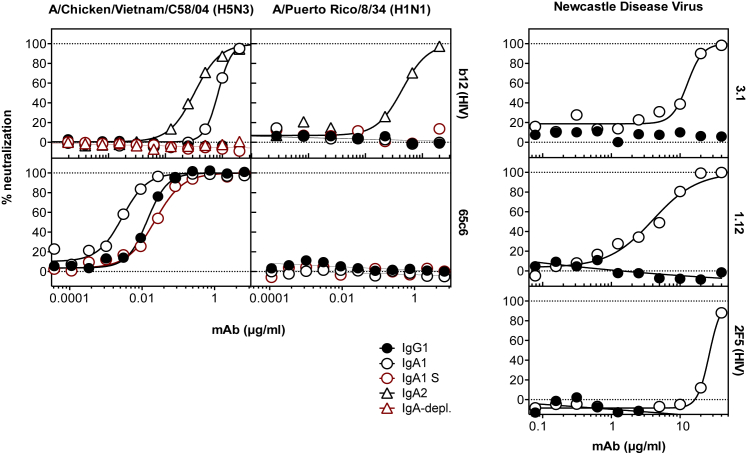
Antiviral Effect of Non-specific IgA Left panels: the antiviral activities of HIV-1 gp120-specific mAb b12 and H5-receptor binding site-specific mAb 65c6 were tested against the indicated viruses. As a control, IgG1, IgA1 expressed in S cells (IgA1 S, red-bordered circles), and samples in which all IgA immunoglobulin has been depleted were used (red triangles). Right panels: antiviral activities of IgG1 or IgA1 of mAbs 3.1, 1.12, and HIV-gp41-specific mAb 2F5 were tested against Newcastle disease virus.

To test whether this receptor-site blockage requires binding of the Fab portion of the antibody, the ability of HIV-1-specific mAbs b12 IgA1 and IgA2 to neutralize rg-A/Chicken/Vietnam/C58/2004 (H5N3) and A/Puerto Rico/8/1934 (H1N1) was tested. Surprisingly, HIV-1 gp120-specific b12 IgA1 was able to neutralize the avian but not the human isolate, while b12 IgA2 was able to neutralize both viruses (Figure 3). This observation is in line with lectin blots demonstrating a predominance of α2–6-linked sialic acid in our IgA_2_ preparation (data not shown). Since neither b12 IgG1 nor b12 IgA1 grown in 293S cells, nor b12 IgA2 samples depleted from antibodies using CaptureSelect beads (IgA-CH1 (Hu)) displayed antiviral activity (Figure 3A), the observed inhibition must originate from the complex sugars of the IgA molecules and not from a contaminant. H5 head-specific mAb C65c6 did not display non-specific neutralization of A/Puerto Rico/8/1934 (H1N1) and only displayed small isotype-dependent changes in activity against rg-A/Chicken/Vietnam/C58/2004 (H5N3) (Figure 3), indicating that this IgA-effect may not apply to all antibodies to the same extent and that Fab binding to the receptor binding site overrides the nonspecific effect. Lastly, to test whether this observation was specific to influenza A viruses, the antiviral activity of influenza-specific mAbs 1.12 and 3.1, as well as of HIV-1 gp41-specific 2F5, was tested against another virus using sialic acid as a receptor—namely, Newcastle disease virus (NDV). As depicted in Figure 3, IgA1, but not IgG1, could neutralize the virus’s infectivity (Figure 3).

**Figure 4:**
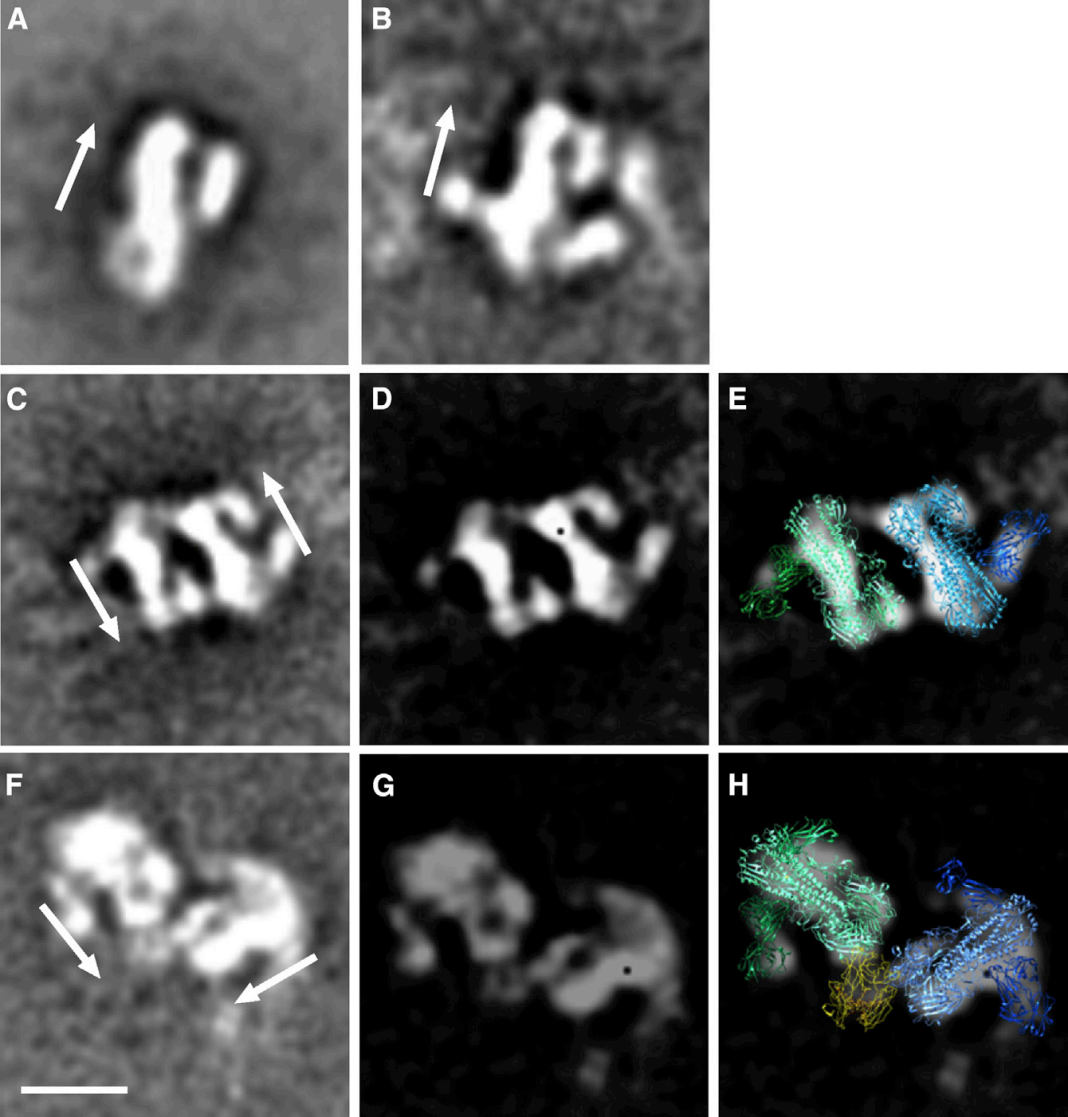
EM Images of HA-Antibody Complexes (A and B) Reference -free class averages of (A) unliganded HA and (B) HA bound to antibody 3.1 IgG1. (C–E) Representative class averages of HA bound to IgG1 (C), filtered image with enhanced contrast (D), and filtered image with docked crystal structures (PDB: 4PY8) in blue and green (E). Two HAs are cross-linked by the two arms of the antibody and are oriented in anti-parallel fashion. (F–H) Representative class averages of HA bound to 3.1 IgA1 (F), filtered image with enhanced contrast (G), and filtered image with docked crystal structures (PDB: 4PY8 and 3CHN) in blue and green (right) (H). Two HAs are oriented so that their head domains are close to one another in a head-to-head fashion. The additional density between the heads likely corresponds to the Fc domain (yellow). Arrows indicate the orientation of the HA, with the head region at the top of the arrow. Scale bar in (F), 10 nm.

To further investigate the potential interaction between the IgA1 and hemagglutinin, we visualized mAb 3.1 bound to recombinant HA from A/Vietnam/1203/2004 (H5N1) by negative stain electron microscopy (EM). The reference-free class averages for HA bound to IgG1 and IgA1 isotypes are shown in Figures 3 and S3. In the majority of class averages in both cases, two HA trimers are crosslinked by the bivalent arms of the antibody. The relative orientation of the two HA trimers to one another differs between the two antibodies: in IgG1-linked dimers, they were predominantly found in opposite orientations, while the IgA1-crosslinked HA trimers were found to be tethered head to head by additional density, which could correspond to CH3 (Figure 4). The HA and Fab components of the complexes were easily identifiable in the class averages, thereby enabling us to dock models of the X-ray structure of HA bound to Fab 3.1 (PDB: 4PY8).

## Discussion

The recombinant antibodies used for this study were monomeric antibodies, while in the respiratory track, IgA is primarily found as dimeric secretory IgA. To rule out the possibility that our 293T-cell-expressed IgA displayed atypical glycosylation, we analyzed the glycosylation of human secretory IgA isolated from the saliva of two individuals by high-pressure liquid chromatography (HPLC). It was found that dimeric IgA contained 14.8%–17.3% terminal sialylation, while monomeric IgA was found to contain between 10.5% and 27.4% terminally sialylated complex glycans. α2–3linked sialic acid made up for 1.4%–6.7% of all complex glycans and was primarily found in multiantennary N-linked glycans (Table 1; Figure S4). The presence of α2–3-linked sialic acid is, therefore, not artificial to our expression system but can also be found on IgA expressed by B cells. However, recombinant 3.1 IgA1 contained more sialic acid than IgA isolated from saliva (Table 1), which may have exacerbated the observed inhibitory effect of recombinant IgA1.

**Table 1:** Predominant Glycosylation Found in IgA Isolated from Human Saliva

Sample	Complex glycans (%)						Oligomannose (%)
	Predominant glycan		Terminal Sialylation		Terminal Galactosylation	Terminal Fucosylation	
	GO	GOF	α2–3	Total			
Patient 1, dimeric IgA	12.2	12.0	1.4	14.8	26.2	56.9	–
Patient 1, monomeric IgA	18.5	5.6	2.9	27.4	36.2	56.6	–
Patient 2, dimeric IgA	12.5	20.7	6.7	17.3	17.4	55.5	–
Patient 2, monomeric IgA	18.8	17.0	5.7	10.5	16.0	56.4	–
3.1 IgA1	43.7	–	30.4	51.0	0.5	1.1	2.1
3.1 IgA2	53.3	–	11.2	22.1	1.2	12.1	1.8
3.1 IgG1	40.3	1.2	–	–	57.2	58.6	–

Different recombinant isotypes of mAb 3.1 as well as IgA from two adult male Caucasians purified from saliva were run on SDS-PAGE to separate dimeric from monomeric forms. The corresponding gel bands were excised, and the N-linked glycans were released from the protein by PNGase digestion and further subjected to digestion with specific glycosylases (EndoH, α2–3,6,8 neuraminidase, β1,4-galactosidase, α-L-fucosidase, β-N-acetylglucosaminidase, α(1-2,3,6)-mannosidase). Differential analysis by HILIC-UPLC was then performed, and the percentages were determined by the sum of the areas under the specific peaks over the area under all peaks.

The lack of dimerization in our expression system is likely to be attributed to the expression system. In contrast to Chinese hamster ovary (CHO) cells, which tend to produce a higher proportion of dimeric IgA (Chintalacharuvu and Morrison, 1999, Moldt et al., 2014), HEK293F or T cells primarily express monomeric IgA (Lorin and Mouquet, 2015). However, as CHO cells are of non-human origin and have been described to produce under-sialylated IgA1 and -2 (Yoo et al., 2010), expression in HE293 cells was preferred for this study. In human serum, IgA is also primarily found as monomeric molecules (Kerr, 1990), indicating that this oligomerization form is biologically common. Moreover, lack of dimerization actually enabled a direct comparison of IgA with IgG1, as their stoichiometries of binding are identical. Last, the available 3D models for secretory IgA1 (Bonner et al., 2009a) and IgA2 (Bonner et al., 2009b) suggest that at least two of the four C-terminal tails are unobstructed by the lateral binding of the secretory component, indicating that, also in higher order complexes, the C-terminal sialic acids would be exposed.

Fab-mediated attachment of the IgA molecules to the conserved stem-epitope in the HA molecule helped to stabilize the low-affinity interaction of sialic acid with HA. However, it was rather unexpected to find that, even with nonspecific mAbs such as b12 or 2F5, this 2- to 4-mM K_d_ affinity interaction (Sauter et al., 1989, Watowich et al., 1994) was able to neutralize the virus. However, for unknown reasons, this was not observed for all antibody/virus combinations. Human influenza A viruses were less sensitive to inhibition by the C-terminal tail glycans than avian isolates, suggesting that they have evolved to overcome this line of defense. Whether this is mediated by the HA protein itself or whether it is due to the sialidase activity of the viral neuramindase remains to be determined. Interestingly, as described earlier, the affinity of HA for sialic acid is very low compared to other attachment/receptor protein interactions that are typically in the nanomolar range; therefore, there seems to be no apparent need for a receptor-destroying enzyme. This notion is further supported by the observation that the neuraminidases of recent human H3N2 isolates actually often fail to release viruses bound to erythrocytes, indicating that their sialidase activity cannot hydrolyze the receptor preferably bound by the HA of the same virus (Gulati et al., 2005). Therefore, we think not only that neuraminidase’s function is to release progeny virus but also that it plays an important role in coping with sialic acids abundantly present in mucosal secretions (Baos et al., 2012, Phalipon et al., 2002). As a matter of fact, many sialylated proteins have been reported to inhibit the influenza A virus (IAV), including human mucin (Baos et al., 2012) and α2-macroglobulin (Pritchett and Paulson, 1989). These inhibitors have been classified to be of the α or γ type, depending on whether the viral neuraminidase was able to hydrolyze the sialic acid moieties or not (“Francis effect”; reviewed in Matrosovich and Klenk, 2003). The observation that a prolonged incubation of mAb 3.1 IgA1 with rg-A/Chicken/Vietnam/C58/2004 (H5N3) abolished the inhibitory advantage of IgA1 indeed suggests that the viral neuraminidase was able to hydrolyze IgA1’s sialic acids (Figure 2E), thus classifying it as an α-type inhibitory protein for this strain. By contrast, heavily tissue-culture- and mouse-adapted A/Puerto Rico/8/1934 (H1N1) appears to have lost this ability.

We, therefore, believe that the glycans of IgA are part of a larger innate defense wall (sugar chainmail) against pathogens using sialic acid as a receptor. However, in contrast to the other proteins, the ability of IgA to attach to virus via the antigen-binding fragment enables it to generate effective local accumulations of sialic acid and can thereby compensate for the poor kinetics of the sialic acid/HA interaction. The importance of IgA’s sialylation is further supported by the observation that, although IgA deficiency is largely asymptomatic in humans, children with this defect are more prone to pseudocroup caused by another sialic-acid binding virus, human parainfluenza 1 virus (Fukushima et al., 2014, Tran et al., 2016). Moreover, the higher sensitivity of avian viruses for this glycan-mediated inhibition could provide an additional innate line of defense to protect humans from acquiring zoonotic infections.

It would have been highly desirable to complement our *in vitro* findings with *in vivo* data; however, passive immunization of mice with human IgA1 is problematic, as it has been shown to rapidly induce elevated tumor necrosis factor alpha (TNF-α) levels and xenotypic antibodies in the recipient (Shi et al., 2011). Mice also only contain one IgA subtype, which is a homolog to human IgA2, and the murine FcαR does not effectively recognize human IgA1 (Balu et al., 2011). The efficacy at which human IgA1 would be able to interact with the murine poly-Ig receptor for transcytosis into mucosal lumen is also hardly defined. Although it has been shown that CH2 glycosylation does not affect binding to FcαR (Gomes et al., 2008, Mattu et al., 1998), IgA sialylation has been shown to affect its ability to activate complement (Nikolova et al., 1994). As murine cells have been shown to produce an IgA glycosylation pattern that is very distinct from that of the human (Yoo et al., 2010), large parts of this study would have to be repeated to characterize the glycan-mediated inhibitory properties of murine or murine-expressed IgA. Alternatively, all approaches to graft the IgA tail on IgG1 ended up with recombinant molecules that did not assemble properly. Also, as reported earlier, removal of the C-terminal glycosylation site alone was not tolerated and resulted in proteins that did not fold properly. Taken together, although highly desirable, a considerable effort is required to establish a small animal system capable of separating the effect of the C-terminal glycosylation from other factors. Moreover, since mice represent a rather artificial experimental system for IAV infection, it is questionable whether these considerable efforts would be justified and actually helped to clarify the importance of the presented findings for human infection.

It has been noted that different B cell lineages have different glycosylation signatures and that these signatures have a substantial impact on the antibodies’ effector functions (Ackerman et al., 2013). For instance, the presence or absence of fucosylation of the N263 glycan improves human IgG1’s ability to interact with FcγRIIIa on natural killer cells (Moldt et al., 2012). Conversely, sialylation of the same glycan lowers its ability to activate complement (Quast et al., 2015). Our findings further strengthen the increasing recognition of the importance of Fc glycosylation on antibody effector functions and suggest that active vaccination aiming at generating heterosubtypic antibody titers could gain effectiveness if the induced antibodies are of the IgA isotype and if adjuvenation could be optimized to induce lineages of highly sialylated IgA-producing B cells. Also, antibodies for passive immunization could gain orders of magnitudes if the advantages of both IgG1 and IgA1 could be combined.

## Experimental Procedures

### Viruses and Cell Lines

Influenza A viruses used for this study were propagated in the amniotic cavity of fertilized hen eggs, except A/FPV/Bratislava/1979 (H7N7) and rg-A/Chicken/Vietnam/C58/2004 (H5N3), which were propagated on Madin-Darby canine kidney (MDCK) cells (discussed later). rg-A/Chicken/Vietnam/C58/2004 (H5N3) is a 6+1+1 reassortant between the internal genes of A/Puerto Rico/8/1934 (H1N1), the neuraminidase from A/Duck/Germany/1215/1973 (H2N3), and a hemagglutinin from A/Chicken/Vietnam/C58/2004 (H5N1), from which the polybasic cleavage site has been removed. GFP-expressing Newcastle disease virus has been propagated as described in Park et al. (2003).

HEK293T and HEK293S (ATCC, CRL-11268 and CRL-3022), both transfectants of the HEK293 cell line (female; ethnicity not available); MDCK cells (ATCC CCL-34, *Canis familiaris*; adult female cocker spaniel); and Vero kidney cells (ATCC CCL-81; *Cercopithecus aethiops*; adult; gender unknown) were propagated in D10 medium (DMEM; ThermoFisher Scientific, Waltham, MA, USA), supplemented with 10% fetal bovine serum (FBS; ThermoFisher Scientific, Waltham, MA, USA), 25,000 U penicillin, 25 mg of streptomycin, and 217 mg GlutaMAX (ThermoFisher Scientific, Waltham, MA, USA) at 37°C/5%CO_2_ according to ATCC’s recommendations. FreeStyle 293-F cells (ThermoFisher Scientific, Waltham, MA, USA) were propagated according to the manufacturer’s instruction.

### Oligonucleotides

The following oligonucleotides have been used for PCR and mutagenesis: GAAA-G-Fw-IgG1-Sall: 5′-tcagcgtcgaccaagggcccatcg-3′; GAAA-G-Rv-CH1-IgG1: 5′-GGGACTTGGTGTGGGTGGGGTaactttcttgtccaccttggtg-3′; GAAA-AAA-Fw Hinge CH3 IgA1: 5′-caccaaggtggacaagaaagttACCCCACCCACACCAAGTCCC-3′; GAAA-AAA-Rv-IgA1-Hindlll: 5′-GGCCAAGCTTTAGTAACAGGTTCCATCC-3′; AGGG-A-Fw-IgA1-Sall: 5′-CCTCAGCGTCGACCCCAAC-3′; AGGG-A-Rv-CH1-IgA1: 5′-gtcacaagatttgggctcTGAAGGCACGGGGCATGG-3′; AGGG-GGG-Fw Hinge CH3 IgG1: 5′-CCATGCCCCGTGCCTTCAgagcccaaatcttgtgac-3′; AGGG-GGG-Rv-IgG1-Hindlll: 5′-caagcttcatttacccggagacag-3′; GGAA-GG-Fw-IgG1-Sall: 5′-tcagcgtcgaccaagggcccatcg-3′; GGAA-GG-Rv-CH1-Hinge IgG1: 5′-AGACAGTCTTGGGTGtgggcatgtgtgagttttgtc-3′; GGAA-AA-Fw-CH2 CH3 IgA 1: 5′-gacaaaactcacacatgcccaCACCCAAGACTGTCTCTGC-3′; GGAA-AA-Rv-IgA1-Hindlll: 5′-GGCCAAGCTTTAGTAACAGGTTCCATCC-3′; AAGG-AA-Fw-IgA1-Sall: 5′-CCTCAGCGTCGACCCCAAC-3′; AAGG-AA-Rv-CH1-Hinge IgA1: 5′-aggtgctgggcacggACAGCAGGATGGGCTTGGTGTTGG-3′; QC-N264D-Rv: 5′-AGGGTGCAAGTCAGGTCTGCTTCACTTCCCAGCAGCAG-3′; QC-N264D-Fw: 5′-GCTGGGAAGTGAAGCAGACCTGACTTGCACCCTGACAGG-3′; QC-N459D-Rv: 5′-GCCATCACGACGCTGACATCGACATGAGTAGGTTTGCC-3′; QC-N459D-Fw: 5′-GGCCGGCAAACCTACTCATGTCGATGTCAGCGTCG-3′.

### Construction of pAbVec Isotype Variants

For the construction of the isotype variants, SalI and HindIII-flanked fragments were synthesized corresponding to the spliced constant regions for IgA1 (GenBank: NC_000014, region 106173505..106175001), IgA2 (GenBank: AK126352), IgG2 (GenBank: NC_000014, region 106109540..106111126), IgG3 (GenBank: K01313), and IgG4 (GenBank: K01316) and were cloned into pAbVec-IgG1-3.1 a SalI-HindIII cassette. For the corresponding 1.12, b12, C65c6, and 2F5 constructs, the AgeIxSalI containing their V-region cassettes were cloned into the isotype variants of the 3.1 containing pAbVec constructs. The sequence of the original pAbVec-IgG1 can be accessed at GenBank: FJ475055.

### Construction of IgA1/IgG1 Chimeric Antibodies

IgG/IgA-chimeric variants of mAbs 1.12 and 3.1 were constructed by fusion PCR using primers listed above. In brief, forward and reverse primers were used in a PCR with pAbVec-IgG1-1.12 and pAbVec-IgA1-3.1 as a template. The resulting fragments were gel purified and fused through the second amplification using forward primer of first fragment and reverse primer of second fragment and Pfu polymerase (Agilent Technologies, Santa Clara, CA, USA). Primers used for amplification of the fragments were: GAAA-G-Fw-IgG1-Sall, GAAA-G-Rv-CH1-IgG1, GAAA-AAA-Fw-Hinge-CH3 IgA1, GAAA-AAA-Rv-IgA1-Hindlll, AGGG-A-Fw-IgA1-Sall, AGGG-A-Rv-CH1-IgA1, AGGG-GGG-Fw-Hinge-CH3 IgG1, AGGG-GGG-Rv-IgG1-Hindlll, GGAA-GG-Fw-IgG1-Sall, GGAA-GG-Rv-CH1-Hinge-IgG1, GGAA-AA-Fw-CH2-CH3-IgA1, GGAA-AA-Rv-IgA1-Hindlll, AAGG-AA-Fw-IgA1-Sall, AAGG-AA-Rv-CH1-Hinge-IgA1, AAGG-GG-Fw-CH2-CH3-IgG1, AAGG-GG-Rv-IgG1-Hindlll, AGAA-AG-Fw-IgA1-Sall, AGAA-AG-Rv-CH1-Hinge IgG1, AGAA-AA-Fw-CH2-CH3-IgA1, AGAA-AA-Rv-IgA1-Hindlll, GAGG-GA-Fw-IgG1-Sall, GAGG-GA-Rv-CH1-Hinge-IgA1, GAGG-GG-Fw-CH2-CH3-IgG1, and GAGG-GG-Rv-IgG1-Hindlll, whereby the first four letters indicate the fusion construct separated by a dash from the fragment and followed by the priming direction and the further designations.

1 μg fusion PCR product and pAbVec-IgG1-1.12 plasmid were digested with Sal l and Hind III, gel purified, quantified, and ligated into the digested backbone using T4 Ligase (New England Biolabs, Ipswich, MA, USA) for 20 min at room temperature (RT). The ligated product was then transformed into 50 μL chemically competent *E. coli*. After sequencing of the mini-preparations, maxi-preparations of positive clones were performed using the PureYield Plasmid Maxiprep System (Promega, Fitchburg, WI, USA). For the generation of the mAb 3.1 variants, the variable region cassette of 1.12 was exchanged for that of 3.1, using SalI and AgeI digestion in all chimeric plasmids.

PCR thermal cycling conditions were as follows: 2 min at 95°C; (20 s at 95°C; 1 min at 68°C; 30 s at 72°C) × 30; 10 min at 72°C; ∞ at 4°C. Fusion PCR thermal cycling conditions were as follows: fragment assembly for chimeric IgG1/IgA1: 5 min at 95°C; 2 min at 72°C (ramping rate [dT] 0.1°C/s); ∞ at 4°C. Amplification was as follows: 2 min at 95°C; (20 s at 95°C; 1.5 min at 68°C; 30 s at 72°C) × 30; 10 min at 72°C; ∞ at 4°C.

### Construction of Glycosylation Variants

N-linked glycosylation consensus sequences located at position 263 of CH2 or 459 of CH3 of the IgA1 Fc were removed from the pAbVec-IgA1-3.1 plasmid by site-directed mutagenesis converting the asparagine of the consensus into aspartic acid. To this end, primer pairs QC-N264D-Rv/QC-N264D-Fw and QC-N459D-Rv,/QC-N459D-Fw and the QuikChange II XL Site-Directed Mutagenesis Kit (Agilent, Santa Clara, CA, USA) were used according to the manufacturer’s instructions. For all other specificities, the AgeI-SalI cassette, containing the V region, was exchanged, as described earlier.

### Recombinant Antibodies

For transfection, either 2.5 × 10^6^ HEK293T or 2.5 × 10^6^ HEK293S cells were seeded into 15-cm tissue culture plates (TPP, Techno Plastic Products, Trasadingen, Switzerland) in 15 mL D10 medium 1 day before transfection. Tissue culture flasks were coated with poly-L-lysin (Sigma-Aldrich, St. Louis, MO, USA) before seeding HEK293S cells. For the transfections, 21.5 μg of the H- and L-chain-expressing plasmids were diluted in 450 μL of 1 M NaCl and adjusted to a final volume of 1 mL with double-deionised water (ddH_2_O). In parallel, 180 μL of 1 mg/mL polyethylenimine (PEI; molecular weight [MW], 25,000; Polysciences, Warrington, PA, USA) were diluted into 1 mL of ddH_2_O. Both solutions were then mixed together, mixed immediately, and incubated at RT for 20 min. 3 mL of the DNA/NaCl/PEI complex solution was then added to each transfection plate. Cells were incubated at 37°C, 5% CO_2_, and 95% humidity for 7 hr before transfection medium was exchanged with 30 mL pre-warmed complete D10. After 6 days of incubation at 37°C/5% CO_2_, supernatants were harvested and clarified by centrifugation at 3,700 relative centrifugal force (rcf) for 15 min and were sterile filtered at 0.2 μm (TPP, Trasadingen, Switzerland). The filter membrane was washed with the same volume of Protein L (ProtL) binding buffer (23 mM NaH_2_PO_4_, 77 mM Na_2_HPO_4_ ⋅ 2H_2_O, 150 mM NaCl [pH 7.2]) and volume adjusted to 1 L with ddH_2_O.

### Antibody Purification

All antibodies were affinity purified using Protein L Agarose Beads (ThermoFisher Scientific, Waltham, MA, USA), according to the manufacturer’s instructions. Abs were eluted by 0.1 M glycine (pH 2.7). The pH was immediately adjusted to a pH of 7 by adding 1 M Tris (pH 8.5). The procedure was repeated once to increase yields.

### Size Exclusion Chromatography

To remove contaminating kappa dimers and free heavy chains, and to perform a buffer exchange, antibodies were additionally purified by size exclusion chromatography. To this end, a Superdex 200 HiLoad 26/60 column (GE Healthcare, Chicago, IL, USA) was equilibrated in PBS before the antibody in elution buffer was loaded, and separation was run at 3 mL/min. Antibody fractions were identified according to their in-line absorbance at 280 nm and used for further analysis. The purity of all fractions was assessed by non-reducing SDS-PAGE, followed by subsequent silver staining. Fractions containing the correctly assembled antibodies were pooled. Since molecular ultrafiltration leads to considerable loss of IgA, pooled antibody fractions were transferred into 3.5-kDa MWCO dialysis tubes (Spectra/Por, Spectrum, Rancho Dominguez, CA, USA) and concentrated via reverse osmosis by sprinkling the tubes with 1–3 g of polyethylene glycol 35000 (Sigma-Aldrich, Buchs, Switzerland).

### Silver Staining of PAGE Gels

Gels were fixed in 100 mL 10% acetic acid, 40% methanol, and 50% ddH_2_O for 25 min. After being washed with water for 25 min, gels were incubated in 50% ethanol for another 25 min. Gels were then incubated in 100 mL ddH_2_O containing 16.7 mg Na_2_S_2_O_3_ for 1.5 min. Gels were washed three times with ddH_2_O and incubated with 0.26 g (w/v) AgNO_3_ and 250 μL of 36% (w/v) formaldehyde for 10 min. After an additional washing step, gels were developed in a solution containing 2 g (w/v) Na_2_CO_3_, 42 μL of a 10% (w/v) Na_2_S_2_O_3_ and 42 μL of 36% (w/v) formaldehyde until the protein bands became visible. The reaction was then stopped by the addition of a 5% (w/v) acetic acid solution.

### IgA2 Depletion

10 μL CaptureSelect IgA-CH1 (Hu) Affinity Matrix (ThermoFisher Scientific, Waltham, MA, USA) was washed two times with 1 mL PBS. The slurry was centrifuged, and the supernatant was discarded. 30 μL of b12 IgA2 (9.32 μg) was incubated with 10 μL prewashed CaptureSelect IgA-CH1 (Hu) Affinity Matrix at RT on a rotor wheel for 1 hr. The mix was then centrifuged at 10,000 rcf for 5 min, and the supernatant was transferred to another 10 μL prewashed beads. The process was repeated a total of four times. 21.9 μL from the depleted b12 IgA2 solution was used for neutralization assays.

### Lectin Blots

To determine the glycosylation pattern, 1 μg/mL reduced Ab was loaded on a 10% SDS-PAGE gel. After separation, proteins were transferred to a nitrocellulose membrane (GE Healthcare, Amersham, UK) using a semi-dry blotting system (Bio-Rad Trans-Blot SD Semi-Dry Transfer Cell, Hercules, CA, USA). Membranes were then blocked with a casein containing western blocking reagent (Roche, 11921673001, Mannheim, Germany) in Tris-buffered saline (TBS; 50 mM Tris-Cl in ddH_2_O [pH 7.6]) overnight. Blots were washed twice with TBS + 0.1% Tween 20 and incubated with a 1:1,000 dilution of biotinylated *Maackia Amurensis* Lectin II (Vector Laboratories; binds α2-3 sialic acid [SA]) in TBS + 0.1% Tween 20 or a 1:1,000 dilution of biotinylated Elderberry Bark Lectin (Vector Laboratories; binds α2-6 SA) in TBS + 0.1 mM CaCl_2_, MgCl_2_, and MnCl_2_ + 0.1% western blot (WB) blocking reagent + 0.1% Tween 20. After washing six times with TBS + 0.1% Tween 20 for 5 min, streptavidin-horseradish peroxidase (HRP) diluted 1:2,000 in TBS was added and incubated for 30 min. Membranes were washed again six times with TBS + 0.1% Tween 20 for 5 min and rinsed once with TBS. Membranes were developed using chemiluminiscent enhanced chemiluminescence (ECL) substrate and a LAS 4000 Mini chemiluminescent image analyzer (ImageQuant, GE Healthcare, Amersham, UK).

### Neuraminidase Treatment

Neuraminidase S (α2-3: New England Biolabs, Ipswich, MA, USA) and neuraminidase A (α2-3,6,8,9: New England Biolabs, Ipswich, MA, USA) were used to desialylate IgA1. To this end, 2 μg IgA variants were combined with H_2_O, to a total volume of 8 μL, and 1 μL 10× Glycobuffer 1 (New England Biolabs, Ipswich, MA, USA). 1 μl neuraminidase was added before the mixture was incubated at 37°C for 1 hr.

### Binding Assay: ELISA

The ELISA 384-well plates (Greiner Bio-One, Kremsmuenster, Austria) were coated with 4 μg/mL recombinant HA from different influenza A viruses in PBS at RT for 1 hr. Plates were then washed 3 times with TBS + 0.1% Tween 20 in an ELx405 Select CW plate washer (BioTek Instruments, Winooski, VT, USA) and blocked with TBS + 0.1% Tween 20 + 5% milk at RT for another hour. Ab variants (20 μg/mL) were diluted 3-fold in TBS containing 0.1% Tween 20 and 0.1% milk, transferred to the plates, and incubated at RT for 1 hr. After washing three times, goat α-human IgK HRP was used as a detection Ab at a dilution of 1:5,000. The 384-well plates were incubated at RT for another hour before they were washed 3 times. To develop the assay, TMB substrate (ThermoFisher Scientific, Waltham, MA, USA) was added. After 2 to 4 min, 2 M H_2_SO_4_ (Sigma-Aldrich, Buchs, Switzerland) was used to stop the reaction. The absorbance at 450 nm was then detected in an EnVision Multiplate Plate Reader (PerkinElmer, Waltham, MA, USA). Prism 6 (GraphPad Software, San Diego, USA) was used to perform non-linear regression of logarithmized data to the Hill Equation.

### Influenza A Virus Neutralization Assay

Each well of a black 384-well plate (Greiner Bio-One, Kremsmuenster, Austria) was seeded with MDCK cells in 50 μL D10 medium. Plates were incubated at 37°C, 5% CO_2_, and 95% humidity overnight. The next day, 3-fold dilutions of the Ab, starting at 40 μg/mL in infectious media, i.e., DMEM supplemented with 0.2% BSA (ThermoFisher Scientific, Waltham, MA, USA), penicillin/streptomycin (Pen/Strep), Glutamax, and 10 mM HEPES (ThermoFisher Scientific, Waltham, MA, USA)], were produced and transferred into a white 384-well plate. Virus diluted to an MOI of 4 was added into the wells with the diluted Ab, resulting in an initial mAb concentration of 20 μg/mL and a MOI of 2. The plates were then incubated at 37°C (5% CO_2_) for 2 hr before the Ab/virus mix was transferred to the corresponding wells of the plates containing the MDCK cells that were washed once with PBS using an ELx405 Select CW plate washer. Infection was then allowed to proceed at 37°C/5% CO_2_ for another hour before the non-adsorbed virus and Abs were washed away with PBS. Then, 50 μL infection media was added to each well, and plates were incubated at 37°C (5% CO_2_) for 6 hr. After incubation, plates were fixed with absolute methanol and stained with 2–4 μg/mL fluorescein isothiocyanate (FITC)-labeled mAb HB65 (ATCC HB-65) in PBS + 1% BSA at 4°C overnight. Cells were washed three times with PBS the next day, and 40 μL PBS was added before fluorescence at 505 nm was determined at 16 locations of each well in an EnVision Multilabel Plate Reader (PerkinElmer, Waltham, MA, USA). The average values from all measurements in a well were then loaded into Prism 6 software (Graphpad Software, San Diego, CA, USA) and normalized against the median values of the control wells, and a non-linear regression of logarithmized data to the Hill Equation was performed.

### Newcastle Disease Virus Neutralization Assay

Six thousand Vero cells (ATCC CCL-81) were seeded in 15 μL medium into each well of a black 384-well plate on the day before the assay. The next day, samples were diluted in a 3-fold dilution series starting from 40 μg/mL in infectious media, which lacked the phenol red dye and only had 5% FBS added. A recombinant Newcastle disease virus expressing GFP with an MOI 4 was added, leading to a final virus concentration of an MOI of 2. After the Ab/virus mix was transferred to cells, then the plates were incubated at 37°C (5% CO_2_) for 24 hr. The fluorescence at 505 nm was determined at 16 locations of each well in an EnVision Multilabel Plate Reader. The average value from all measurements in a well were then loaded into Prism 6 software and normalized against the median values of the control well, and a non-linear regression of logarithmized data to the Hill Equation was performed.

### Hemagglutination Inhibition Assay

To determine the optimal virus-to-erythrocyte ratio, 50 μL 2-fold virus stock dilutions were prepared in V-shaped 96-well plates (Greiner Bio-One, Kremsmuenster, Austria). The same volume of a 1% chicken red blood cell (CRBC; Bell Food Group, Basel, Switzerland) suspension was then added to each well and incubated at RT for 30 to 60 min until CRBC pellets had formed in the negative control.

After quantifying the optimal virus-to-red blood cell (RBC) concentration, 50 μL of serial 2-fold dilutions of mAbs were prepared, starting at a concentration of 200 μg/mL, and mixed with 50 μL of the optimal virus dilution. After 30-min incubation at 4°C, 50 μL of a 0.5% CRBC suspension were added to all columns, resulting in a maximal mAb concentration of 25 μg/mL. Plates were incubated at RT until CRBC pellets could be observed in the negative control.

### Negative Stain EM

Antibody/HA complexes were formed by incubating 50 μg recombinant HA from A/Vietnam/1203/2004 (H5N1) with 150 μg of antibody in PBS at 4°C overnight. High-molecular-weight complexes were then isolated by size exclusion chromatography on a Superdex 200 Increase 10/300 GL column (GE Healthcare, Amersham, UK). Complexes were then added onto carbon-coated copper mesh grids and stained with 1% uranyl formate for 30 s. Samples were imaged on a Tecnai F12 microscope using the automated image acquisition software Leginon (Suloway et al., 2005), and images were acquired on a Tietz 4k CMOS detector at a magnification of 52,000× and a final pixel size of 2.05 Ǻ. Raw images were automatically uploaded into the Appion database (Lander et al., 2009). Particles were individually picked from raw images using DoG Picker (Voss et al., 2009), placed into a stack, and binned by 2. Reference-free two-dimensional classification was done using multi-reference alignment/multivariant-statistical analysis (MRA/MSA) (van Heel et al., 1996). Class averages that did not correspond to HA were removed from subsequent analyses. Raw particle picks from a clean stack were then subjected to two-dimensional classification by iterative stable alignment and clustering (ISAC) (Yang et al., 2012). UCSF Chimera (Pettersen et al., 2004) was used to dock crystal structures and for figure generation.

### Purification of IgA from Human Saliva

For the purification of human IgA, two male Caucasian volunteers aged between 30 and 45 years collected between 30 and 35 mL of saliva by repetitive spitting into a 50-ml tube on ice. Saliva was then clarified by centrifugation at 4,000 rpm and 4°C for 20 min. 20 mL of the clarified saliva was mixed with 20 mL of ProtL binding buffer. Saliva IgA was then affinity purified via 1 mL of Protein L slurry and buffer exchanged as described earlier.

### Enzymatic Release and Fluorescent Labeling of N-Linked Glycans

The secretory IgA samples isolated from saliva of two individuals were fractionated by SDS-PAGE Novex NuPAGE Bis-Tris 4%–12% pre-cast gels (Life Technologies, Paisley, UK). After being alternately washed with acetonitrile and water for five times, the excised gel bands were incubated with protein N-glycosidase F (PNGase F; 500 U/mL; New England Biolabs, Hertfordshire, UK) in a reaction buffer (50 mM NaHCO_3_ [pH 7.4]) at 37°C for 16 hr. The released glycans were extracted from the gel matrix by washing three times with water and then dried in a SpeedVac Concentrator Plus (Eppendorf, Stevenage, UK). The glycan samples were resuspended in 30 μL water, followed by incubation at 80°C for 1 hr in 80 μL labeling mixture containing 3% (w/v) 2-aminobenzoic acid (2-AA), 4.5% w/v sodium cyanoborohydride, 4% w/v sodium acetate trihydrate, and 2% w/v boric acid in methanol. Removal of excess label from the 2-AA fluorescently labeled glycan samples was performed using Speed Amide-2 cartridges (Applied Separations, Allentown, PA).

### Exoglycosidase Sequencing of N-Linked Glycans

The 2-AA labeled glycans were sequentially digested using the following exoglycosidases according to the manufacturers’ instructions: α2-3,6,8 neuraminidase from *Clostridium perfringens* (New England Biolabs, Hertfordshire, UK), α2-3 neuraminidase from *Streptococcus pneumoniae* (New England BioLabs, Hertfordshire, UK), β1,4-galactosidase from *Bacteroides fragilis* (New England Biolabs, Hertfordshire, UK), and α-L-fucosidase from bovine kidney (Sigma-Aldrich, Dorset, UK), β-*N*-acetylglucosaminidase from *Xanthomonas manihotis* (New England Biolabs, Hertfordshire, UK), and α(1–2,3,6)-mannosidase from Jack bean (Sigma-Aldrich, Dorset, UK). Endoglycosidase H (endoH) from *Streptomyces picatus* (New England BioLabs, Hertfordshire, UK) was used for quantification of oligomannose structures. Prior to chromatographic analysis, polyvinylidene difluoride (PVDF) protein-binding membrane plates (Millipore, Feltham, UK) were used for the removal of enzymes.

### Chromatographic Analysis of N-Linked Glycans

Analysis of the fluorescently labeled N-linked glycans was performed using hydrophilic interaction chromatography-ultra-performance liquid chromatography (HILIC-UPLC), with the 2-AA-labeled glucose homopolymer ladder (Ludger, Abingdon, UK) being used as a calibration standard. The following gradient was run on a 2.1 mm × 10 mm (1.7-μm particle size) ACQUITY Ethylene Bridged Hybrid (BEH) glycan column (Waters, Elstree, UK) pre-installed in the Waters ACQUITY UPLC instrument: time = 0 min (t = 0): 22% A, 78% B (flow rate of 0.5 mL/min); t = 38.5: 44.1% A, 55.9% B (0.5 mL/min); t = 39.5: 100% A, 0% B (0.25 mL/min); t = 44.5: 100% A, 0% B; and t = 46.5: 22% A, 78% B (0.5 mL/min), where solvent A was 50 mM ammonium formate (pH 4.4) and solvent B was acetonitrile. Fluorescence was measured using an excitation wavelength of 250 nm and a detection wavelength of 428 nm. Data processing was performed using Empower 3 software. The percentage of abundance of oligomannose- and that of complex-type glycans were calculated by integration of the relevant peak areas before and after EndoH digestion, following normalization.

## Supplemental Information

Document S1. Figures S1–S4

Movie S1. Visualization of a Potential Immune Complex Formation in which the C-Terminal Glycans of IgA1 3.1 Are Brought into Proximity of the HA Receptor Binding Site, Related to Figure 4 For the generation of the animation, a single IgA1 molecule was extracted from the structure for human secretory IgA1 (PDB: 3CHN), and each IgA1 Fab region was structurally aligned with one Fab of the 3.1xH1 complex (PDB: 4PY8) in MacPyMol 1.8. The molecules were colorized (IgA1 orange, 3.1 Fab blue, HA1 green, HA2 gray, receptor binding site red, N459 purple) and the protein surface meshes were exported as a VRML2 file. Following import into Blender 2.78, meshes were scaled by a factor of 0.025, cleaned from duplicate vertices and additional imported features such as viewpoints and lights. For illustration purpose, glycan 156 from PDB 5T3x was exported as VRLM2 file, imported into Blender 2.78 as described above, duplicated, and visually placed at both N459 residues. For the animation, a rigid 5-bone armature (1x Fc, 2x hinge, 2x Fab) with joints at either end of the hinge region (in proximity of proline 226 serine 240) was generated and skinned with the imported protein surface meshes using automatic weighting. After manually adjusting the weighting and illuminating the scene by two light emitting planes, the animation was defined by two keyframes: the first keyframe defining the original posture of the armature at frame 30, and a second keyframe defining the posture in which the HA receptor binding sites are in proximity to the glycans at frame 200. Blender 2.78 automatically calculated the transition between both keyframes. Please note that this animation is for spacial illustration purposes only, and does not take steric hindrance by Olinked glycosylation of the hinge into account. Also, it does not represent the complexes seen in Figure 4 as those are likely to be *in trans* interactions between different complexes.

Document S2. Article plus Supplemental Information
