# Supplementary material for: Glycosylation of Human IgA Directly Inhibits Influenza A and Other Sialic-Acid-Binding Viruses
Source: Cell Rep. 2018 Apr 5;23(1):90–9. doi: 10.1016/j.celrep.2018.03.027 (PMC5905402; doi:10.1016/j.celrep.2018.03.027)
Supplement: Document S1. Figures S1–S4 [file mmc1.pdf]

Cell Reports, Volume *23*

Supplemental Information

Glycosylation of Human IgA Directly Inhibits Influenza A and Other Sialic-Acid-Binding Viruses

Michael A. Maurer, Larissa Meyer, Matteo Bianchi, Hannah L. Turner, Ngoc P.L. Le, Marco Steck, Arkadiusz Wyrzucki, Vanessa Orlowski, Andrew B. Ward, Max Crispin, and Lars Hangartner

Supplementary Material

Supplementary Figure 1

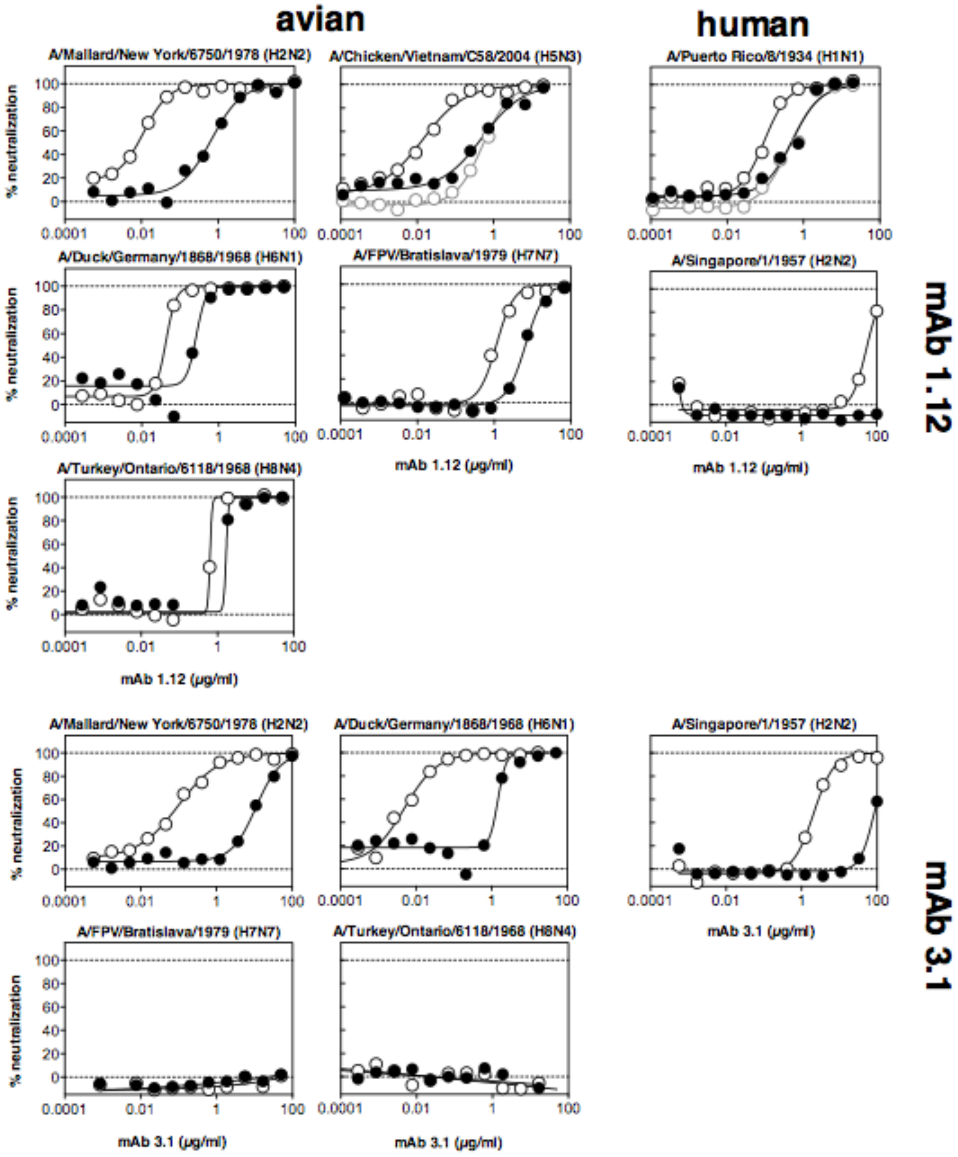

Suppl. Figure 1: Comparison of the neutralizing activity of mAb 3.1 and 1.12 expressed as different isotypes. Neutralizing activity of the indicated antibody expressed as IgA1 (black open face) and IgG1 (black bold face) isotype against the indicated virus. While mAb 3.1 only neutralized viruses from phylogenetic group A, mAb 1.12 neutralizes viruses from both phylogenetic groups. In some experiments, IgA1 expressed in 293S cells (gray open face) was included, as well.

Supplementary figure 2

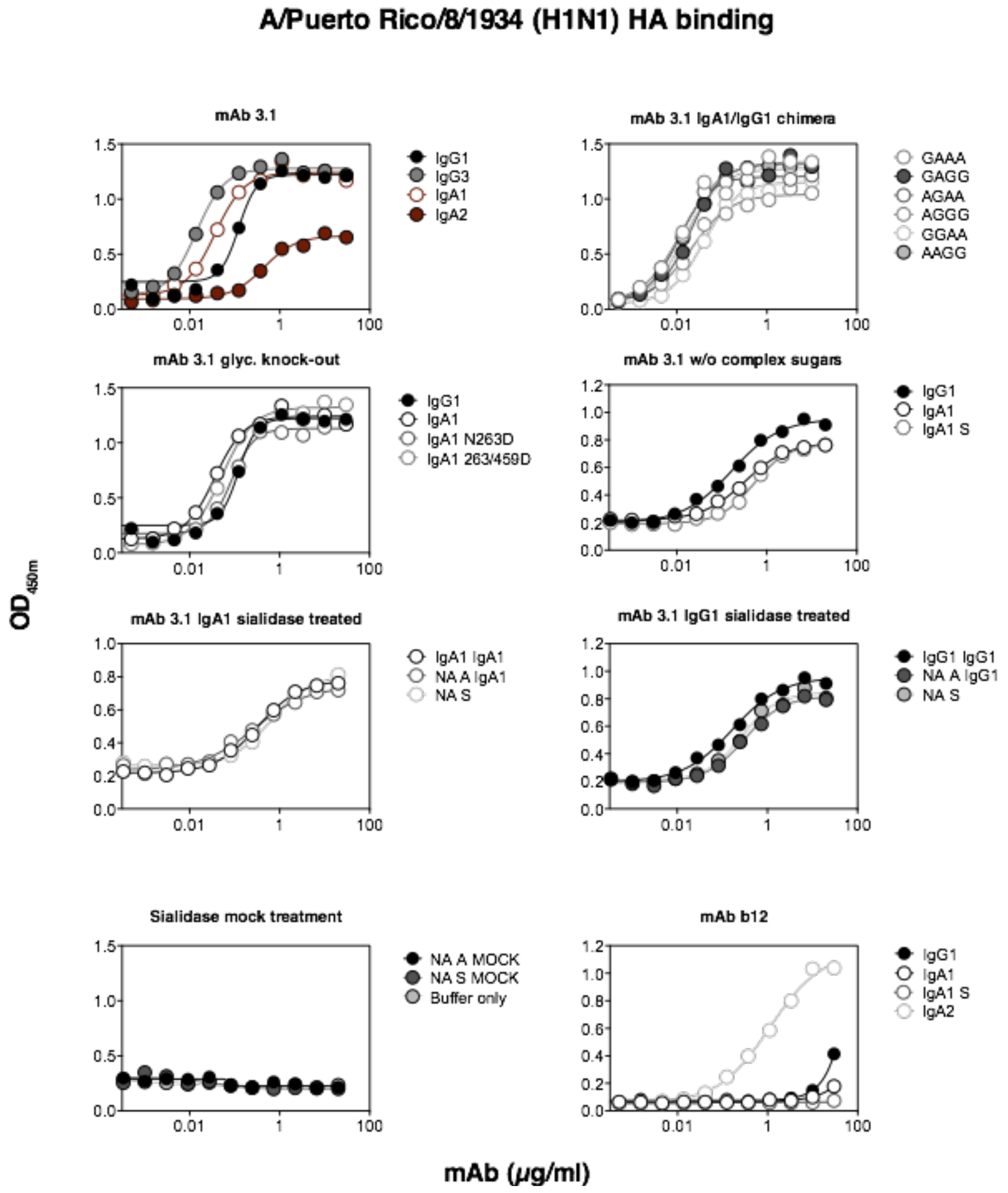

Suppl. Figure 2: Comparison of binding to recombinant HA from A/Puerto Rico/8/1934 (H1N1) by ELISA. Binding of the indicated antibodies was assessed and detected with an appropriate HRPO-labeled secondary antibody. No differences in binding could be detected for all antibodies used for this study. The low plateau for IgA2 could be attributed to a poorer detection of IgA2 by the secondary antibody used.

Suppl. Figure 3

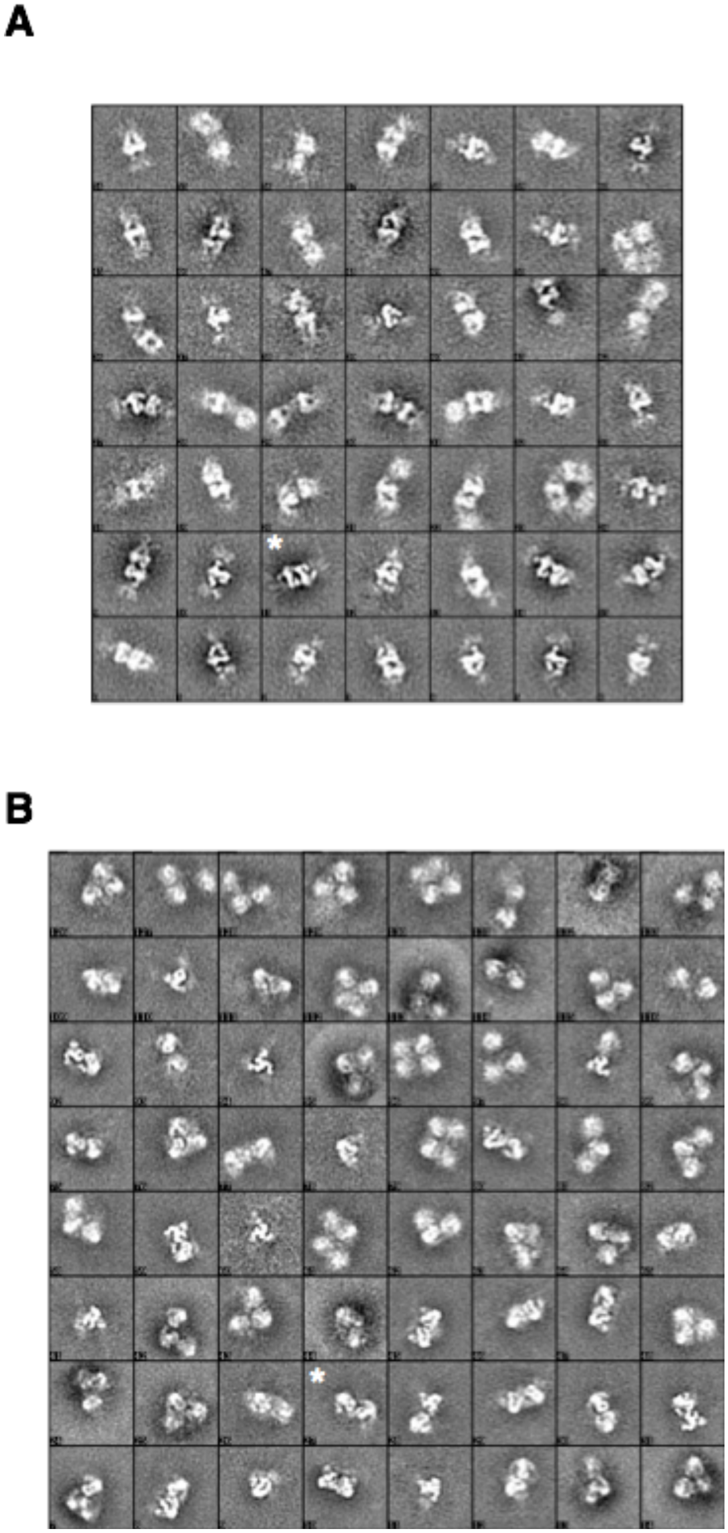

Suppl. Figure 3: Reference free 2D class averages of HA bound to IgG1 (A) and IgA1 (B). The panels used for Figure 4 are indicated by a white asterisk.

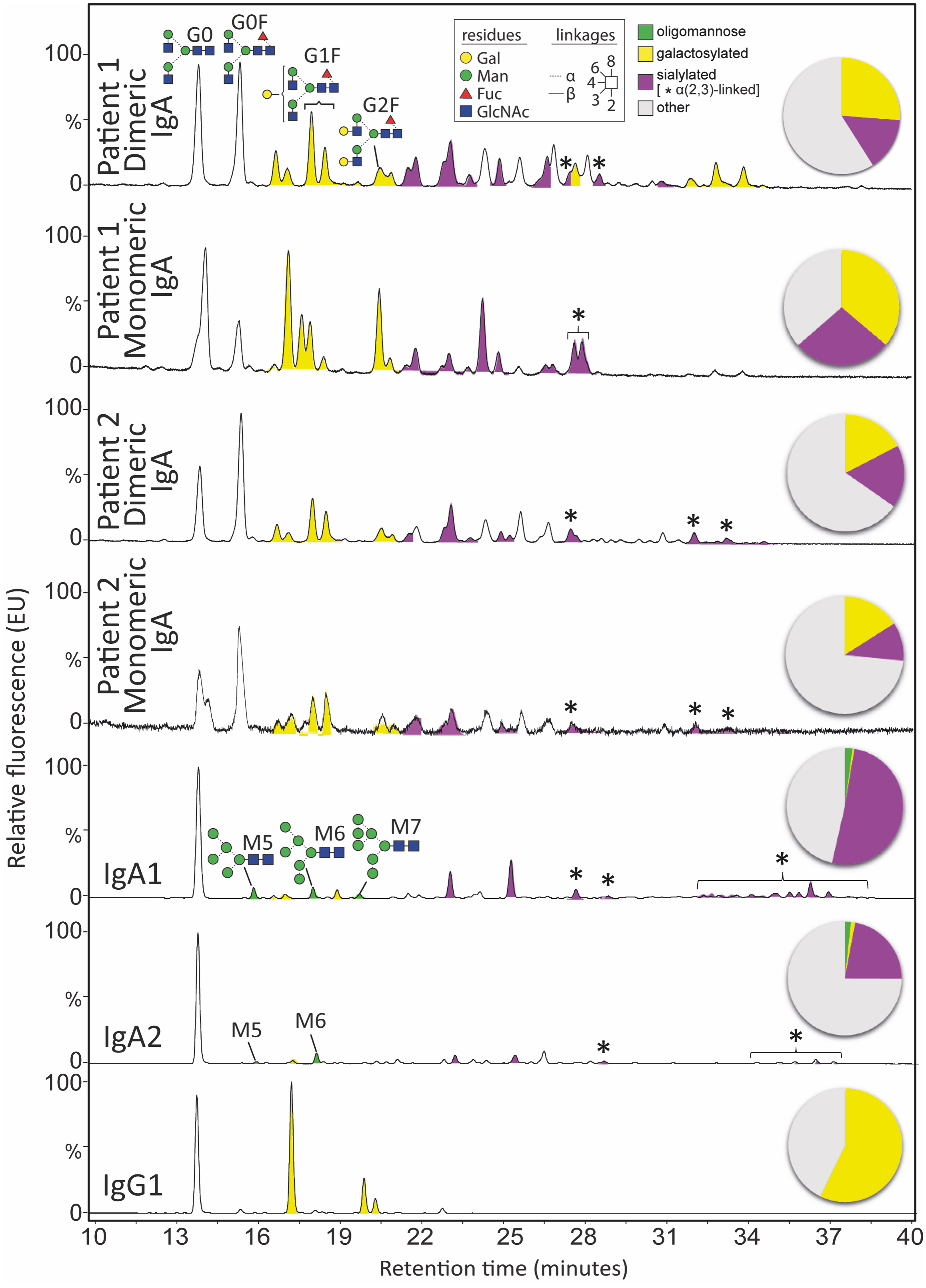

Suppl. Figure 4: High-resolution UPLC traces of glycans released from the indicated antibody preparation
